# Characteristics of people with epilepsy in three Eastern African countries – a pooled analysis

**DOI:** 10.1186/s12883-022-02813-z

**Published:** 2022-08-26

**Authors:** Dominik Stelzle, Joyce Kaducu, Veronika Schmidt, Tamara M. Welte, Bernard J. Ngowi, William Matuja, Gabrielle Escheu, Peter Hauke, Vivien Richter, Emilio Ovuga, Bettina Pfausler, Erich Schmutzhard, Action Amos, Wendy Harrison, Luise Keller, Andrea S. Winkler

**Affiliations:** 1 Center for Global Health, Department of Neurology, School of Medicine, Technical University of Munich, Ismaninger Strasse 22, 81675 Munich, Germany; 2 Ministry of Health, Kampala, Republic of Uganda; 3 Centre for Global Health, Institute of Health and Society, University of Oslo, Oslo, Norway; 4 Department of Neurology, University Hospital Erlangen, Erlangen, Germany; 5 National Institute for Medical Research, Muhimbili Medical Research Centre, Dar es Salaam, Tanzania; 6 University of Dar Es Salaam, Mbeya College of Health and Allied Sciences, Mbeya, Tanzania; 7 Department of Neurology, Muhimbili University of Health and Allied Sciences, Dar es Salaam, Tanzania; 8 Department of Neurology, Kliniken Ostallgaeu-Kaufbeuren, Kaufbeuren, Germany; 9 Department of Radiology, University Hospital Tuebingen, Tuebingen, Germany; 10 Department of Mental Health, University of Gulu, Gulu, Uganda; 11 Department of Neurology, Medical University of Innsbruck, Innsbruck, Austria; 12 National Epilepsy Association Malawi, International Bureau of Epilepsy, Lilongwe, Malawi; 13 Department of Infectious Disease Epidemiology, Imperial College London, London, UK; 14 Department of Neurology, School of Medicine, Technical University of Munich, Munich, Germany

**Keywords:** Epilepsy, Sub-Saharan Africa, Global health, Treatment gap

## Abstract

**Background:**

Epilepsy is one of the most common neurological disorders worldwide. Yet, its treatment gap is large in some areas and especially in sub-Saharan Africa data on clinical, radiological and semiological characteristics, as well as on treatment of persons with epilepsy (PWE) are still scarce.

**Methods:**

We pooled data from four cross-sectional studies on epilepsy in eastern Africa. Two studies from Malawi and Uganda were community-based; two studies in Tanzania (urban Dar es Salaam and rural Haydom) were hospital-based. Clinical characteristics of PWE were assessed by the same questionnaire. Additionally, data on treatment were collected and computed tomography (CT) scans were performed.

**Results:**

Overall, 1179 PWE were included in our analysis (581 (49.3%) female, median age 22 years (IQR 15–32 years)). Up to 25% of the patients had focal onset seizures. Those showed a higher rate of remarkable CT scan findings, with especially post-ischaemic and neurocysticercosis-associated lesions, compared to PWE with generalized onset seizures (35.1% vs. 20%). The majority of the patients experienced tonic–clonic seizures (70–85%). Only 67–78% of PWE received anti-seizure medication (ASM) treatment in the community-based studies, mostly monotherapy with phenobarbital, phenytoin or carbamazepine. Yet, underdosage was frequent and a large proportion of PWE received alternative non-ASM treatment consisting of herbal treatment (up to 83%) and/or scarification (up to 20%).

**Conclusions:**

Epilepsy is common in sub-Saharan Africa, often caused by neurocysticercosis or ischaemic strokes. PWE suffer from high seizure rates and subsequent injuries, as well as from socio-economic consequences due to insufficient ASM treatment. This pooled analysis illustrates the need for structural programmes for adequate identification, education, assessment and treatment of PWE in sub-Saharan Africa.

**Supplementary Information:**

The online version contains supplementary material available at 10.1186/s12883-022-02813-z.

## Key points

- Epilepsy is common in low-income and middle-income countries and aetiologies seem to differ from high-income countries
- Neurocysticercosis and post-ischaemic lesions are common CT findings among people with epilepsy in eastern Africa
- Many people with epilepsy are either not on anti-seizure medication or are underdosed resulting in high seizure frequency
- Carbamazepine, Phenobarbital, and Phenytoin are commonly used anti-seizure medication whereas Valproic acid is only rarely used
- Injuries due to epileptic seizures are common in eastern Africa and may reflect the high seizure-related mortality

## Background

Epilepsy is affecting approximately 46 million people globally and therefore is one of the most common neurological disorders worldwide [1]. If one accounts for family members and relatives of persons with epilepsy (PWE), whose lives are likewise socially and economically affected, this number will increase considerably. The majority of PWE are suspected to live in low-income and middle-income countries (LMIC) [2]. However, epilepsy prevalence estimates are varying notably, probably due to a wide range of different aetiologies including genetic, environmental and behavioural risk factors. *Taenia solium* neurocysticercosis (NCC) is known to be one of the leading causes of epilepsy in LMIC [3]. However, there is still a large research gap concerning the contribution of other infectious diseases like cerebral malaria, meningitis or HIV/AIDS to the prevalence of epilepsy in low-resource settings. Same applies for the raising burden of cerebrovascular diseases due to the epidemiological transition in many LMIC [4–8].

As neuroimaging, electroencephalography (EEG) or direct observations of epileptic seizures are rare, diagnosis of epilepsy mostly is made based on reports and descriptions of epileptic events by the patients themselves or observers. Consequently, reliable estimates on epilepsy prevalence and causes are difficult to ascertain. Furthermore, other medical conditions like syncope, movement or sleep disorders may be difficult to distinguish from epileptic seizures, especially by non-specialists. Additionally, underreporting may occur due to potential stigma and discrimination of PWE, especially in rural areas [9–11].

Despite cheap and effective anti-seizure medication (ASM) being available, and often being free of charge, access to sustained medical treatment is limited for many people in LMIC [12]. Large treatment gaps were already reported before, i.e. PWE not being treated with ASM or treated with insufficient dosage, resulting in up to more than 60% of PWE in Africa not receiving adequate treatment [13–16]. Reasons are partly patient-related, particularly concerning cultural beliefs and stigma about epilepsy; but also financial constraints from both a patient and a system perspective, or stock-out of ASM play an important role [11, 17, 18].

Knowledge about geographic, demographic, and clinical characteristics, types and frequency of epileptic seizures, relevant co-morbidities as well as the underlying causes of epilepsy is crucial for good clinical practice and appropriate management of PWE as well as adequate planning of resource allocation. That is why this study aimed to pool these factors from four studies on epilepsy in three African countries.

## Methods

Characteristics and methodology of this pooled analysis have been described elsewhere [19].

In short, the current study was part of a large study that focused on neurocysticercosis in PWE from four study sites of three eastern sub-Saharan African countries. Here we report on the pooled epilepsy data. PWE (*n* = 1179) were examined in-depth including demographic, clinical, semiological (related to epileptic seizures), neuroradiological and therapeutic (related to anti-seizure medication) variables. Two of the four studies were conducted in hospital-based settings in Tanzania (Haydom and Dar es Salaam) [3, 20]. The other two studies were community-based; one was conducted in northern Uganda and one in Balaka district in Malawi.

**Fig. 1:**
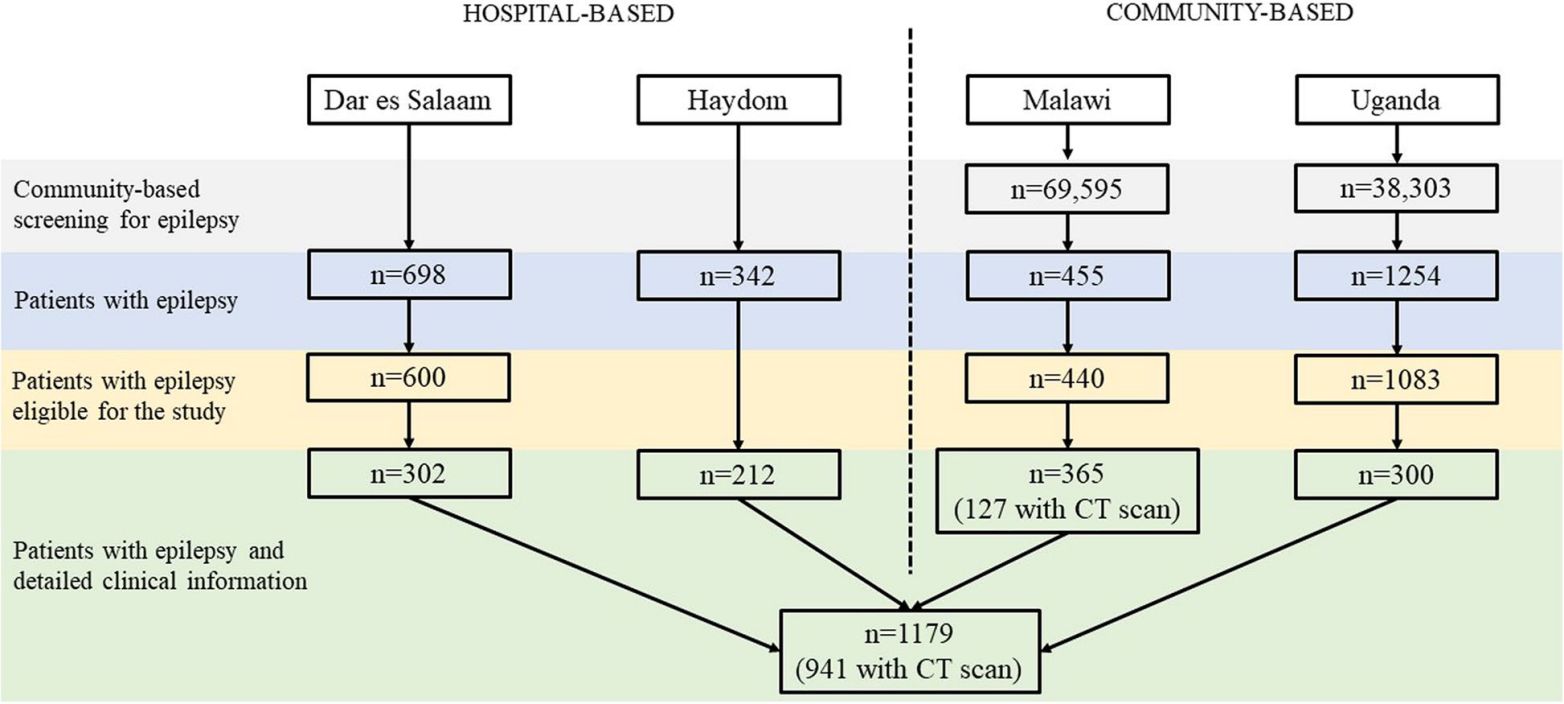
Flowchart of the patient selection by site

A flowchart of included patients by site is displayed in Fig. 1 and the detailed methodology can be found in the Supplement.

### Evaluation of epilepsy

All four studies applied the same in-depth epilepsy questionnaire, despite few questions not included in all studies (indicated not applicable [NA] in the tables). Data collection comprised information on demography, seizure history and semiology, pre-existing health conditions, past medical and psychiatric history, perinatal and family history, ASM and neurological examination. CT scanning was performed locally at the different study sites, under supervision of trained clinical stuff. CT reports were all done by the same neuroradiologist (VR) at the Department of Radiology, Technical University of Munich (TUM), Germany.

Semiology of seizure type was categorized according the 2017 ILAE definition of seizure onset (focal/generalised) [21]. Diagnosis was based on reported semiological characteristics only. Consequently, report of an aura before seizures or an unilateral beginning led to classification as focal onset seizure, whereas lack of these signs led to classification as generalised onset seizure. We additionally used classification according to Winkler et al. [22], who proposed an epilepsy definition which was created for African settings. For this classification we used all information available (apart from CT scan).

### Statistical analysis

Presentation of baseline data was done by centre and as total values. For binary values, numbers and proportions are presented; median and interquartile range (IQR) are presented for continuous data. Although screening and examination tools were well comparable between studies, we mostly did not perform statistical testing because of different recruitment procedures and potential selection bias. R version 4.1.1. was used for all analyses.

## Results

Overall, 2749 PWE were included in the four studies. In the community-based studies, epilepsy prevalence was 1.1% (95%CI 1.0–1.1%) in Malawi and 3.3% (95%CI 3.1–3.4%) in Uganda (Table 1). After exclusion of patients who were not eligible for further clinical work-up, 1179 patients remained. In Tanzania 514 (hospital-based; Dar es Salaam: 302, Haydom: 212), in Malawi 365 (community-based) and in Uganda 300 (community-based). For more details refer to Fig. 1. Of the 1179 PWE, 581 (49.3%) were female, and median age of patients was 22 years (IQR 15 to 32 years). Patients from Malawi were in the median younger (18 years [IQR 12 to 31 years]) than patients in the other three studies. In the urban Dar es Salaam study site, more patients were single than in the other rural study sites. In Haydom and Uganda, around 90% of patients were Christians; in Dar es Salaam and Malawi the percentage was smaller (51% and 58%) and there were proportionally more Muslims included. 941 (80%) of the 1179 patients had a CT scan (Table 1).

**Table 1:** Baseline characteristics of persons with epilepsy from four different studies

		**Tanzania** **(Dar es Salaam)**	**Tanzania** **(Haydom)**	**Malawi**	**Uganda**	**Overall**	**Overall (with CT available)**
		**Urban**	**Rural**	**Rural**	**Rural**		
		**Hospital-based**	**Hospital-based**	**Community-based**	**Community-based**		
n		302	212	365	300	1179	941
Epilepsy prevalence (95%CI)				1.1% (1.0–1.3%)	3.3% (3.1–3.4%)		
Sex	female	160 (53.0)	104 (49.1)	189 (51.8)	128 (42.7)	581 (49.3)	460 (48.9)
	male	142 (47.0)	108 (50.9)	176 (48.2)	172 (57.3)	598 (50.7)	481 (51.1)
Age group	children (< 15 years)	58 (19.2)	42 (19.8)	103 (29.3)	57 (19.0)	260 (22.3)	203 (21.6)
	adolescents (15–29 years)	148 (49.0)	113 (53.3)	141 (40.2)	154 (51.3)	556 (47.7)	462 (49.1)
	adults (30–49 years)	73 (24.2)	38 (17.9)	77 (21.9)	61 (20.3)	249 (20.6)	194 (20.6)
	elderly (≥ 50 years)	23 (7.6)	19 (9.0)	30 (8.5)	28 (9.3)	100 (8.6)	82 (8.7)
Age in years	median (IQR)	23 [16–32]	22 [15–30]	21 [13–32]	21 [15–32]	22 [15–32]	22 [15–32]
Marital status	single	232 (76.8)	126 (59.4)	232 (63.6)	58 (19.3)	648 (54.9)	226 (24)
	married/cohabiting	54 (17.9)	81 (38.2)	92 (25.2)	55 (18.3)	282 (23.9)	51 (5.4)
	seperated/divorced/widowed	14 (4.6)	5 (2.4)	38 (10.4)	24 (8)	82 (6.9)	497 (52.8)
	unknown	2 (0.7)	0 (0)	3 (0.8)	163 (54.3)	168 (14.2)	167 (17.7)
Religion	Christian	151 (50)	189 (89.2)	215 (58.9)	284 (94.7)	839 (71.2)	707 (75.1)
	Muslim	148 (49)	3 (1.4)	147 (40.3)	6 (2.0)	304 (25.8)	199 (21.1)
	other	0 (0.0)	6 (2.8)	3 (0.8)	10 (3.3)	19 (1.6)	18 (1.9)
	unknown	3 (1.0)	14 (6.6)	0 (0.0)	0 (0.0)	17 (1.4)	17 (1.8)
Occupation	farmer	0 (0)	196 (92.5)	179 (49.0)	91 (30.3)	466 (39.5)	334 (35.5)
	professional	59 (19.5)	0 (0)	7 (1.9)	3 (1)	69 (5.9)	66 (7.0)
	student	93 (30.8)	0 (0)	103 (28.2)	0 (0)	196 (16.6)	149 (15.8)
	trader/business	3 (1)	5 (2.4)	15 (4.1)	9 (3)	32 (2.7)	20 (2.1)
	other	15 (5)	8 (3.8)	27 (7.4)	197 (65.7)	247 (20.9)	226 (24.0)
	none	123 (40.7)	3 (1.4)	14 (3.8)	0 (0)	140 (11.9)	134 (14.2)
	unknown	9 (3)	0 (0)	20 (5.5)	0 (0)	29 (2.5)	12 (1.3)
Pork consumption		155 (51.7)	162 (77.5)	103 (29)	213 (71.2)	637 (54.0)	568 (61.1)
Family pork consumption		184 (61.3)	87 (41.0)	NA	NA	271 (52.9)	271 (52.9)
Free roaming pigs living close by		23 (7.7)	NA	58 (16)	86 (28.8)	167 (14.2)	134 (18.6)
Latrine use		221 (90.6)	203 (97.1)	NA	220 (73.6)	644 (85.6)	644 (85.6)

### Characteristics and treatment of patients with epilepsy

Clinical characteristics are presented in Table 2. Median age at first epileptic seizure ranged from 10 to 15 years between the studies. Frequency of seizures varied between the studies. Whilst 77.1% of patients in Haydom had seizures only once a year or less often, this proportion was 4.6% in Malawi, 21.0% in Dar es Salaam and 21.5% in Uganda. Yet, up to 17.5% of PWE (in Malawi) experienced seizures daily to weekly. In Uganda and Malawi, every other PWE had ever experienced injuries during fits. These were mostly tongue bites, bruises or burns. The majority of patients at all sites experienced tonic–clonic seizures (from 70 to 85%; Table 2). According to the ILAE criteria for seizure type, 7.7% of patients in Uganda had focal onset seizures, 20.2% in Dar es Salaam, 21.1% in Malawi and 28.8% in Haydom. However, the vast majority of PWE had generalised onset seizures according to the ILAE criteria. Most PWE had generalised seizures within a specific age range according to the definition by Winkler et al., which represents the category in which least focal onset seizures are assumed [22].

**Table 2:** Characteristics of persons with epilepsy from four different studies

		**Tanzania (Dar es Salaam)**	**Tanzania (Haydom)**	**Malawi**	**Uganda**
n		302	212	365	300
Loss of consciousness		300 (99.3)	210 (99.1)	359 (98.4)	287 (95.7)
Motor activity	tonic–clonic	211 (69.9)	178 (84.0)	311 (85.2)	213 (71.0)
	tonic	31 (10.3)	25 (11.8)	21 (5.7)	58 (19.3)
	clonic	12 (4.0)	9 (4.2)	25 (6.8)	15 (5.0)
	myoclonic	0	0	0	5 (1.7)
	eye rolling/teeth grinding only	19 (6.3)	0	3 (0.8)	9 (3.0)
	no movements at all	29 (9.6)	0	5 (1.4)	0
Motor activity: side of body	bilateral	239 (79.9)	179 (84.4)	249 (75.9)	283 (94.3)
	unilateral	31 (10.4)	33 (15.6)	74 (22.6)	17 (5.7)
	none	29 (9.7)	0	5 (1.5)	0
	Information missing	3	3	37	0
Other signs observed (froth from the mouth, urine/stool incontinence)		246 (81.5)	199 (93.9)	323 (88.5)	287 (95.7)
Injuries during seizures		56 (18.5)	86 (40.6)	204 (56.2)	150 (50.0)
	face/head	22/46 (47.8)	29/82 (35.4)	83/177 (46.9)	94/149 (63.1)
	thorax	1/46 (2.2)	3/82 (3.7)	49/177 (27.7)	7/149 (4.7)
	limbs	16/46 (34.8)	58/82 (70.7)	45/177 (25.4)	81/149 (54.4)
	Information missing	10	4	27	1
Treatment for injuries		34 (65.4)	NA	56 (27.5)	105 (70.5)
	operation	3/31 (9.7)	NA	1/22 (4.5)	3/105 (2.9)
	stitching	4/31 (12.9)	NA	0/22	0/105
	dressing	27/31 (87.1)	NA	20/22 (90.9)	81/105 (77.1)
	medication	4/31 (12.9)	NA	15/22 (68.2)	14/105 (13.3)
	other	1/31 (3.2)	NA	0/22	7/105 (6.7)
	information missing	3	NA	34	0
Reorientation after seizures		285 (95.0)	210 (99.5)	335 (91.8)	201 (67.0)
Age seizures started (years)	median (IQR)	15 (11–22)	15 (11–23)	10 (6–20)	14 (11–23)
	younger than 21	222 (74.0)	152 (72.0)	237 (77.5)	218 (72.7)
	older than 21	78 (26.0)	59 (28.0)	82 (22.5)	82 (27.3)
Frequency of seizures	daily to weekly	44 (14.9)	0	57 (17.5)	9 (4.1)
	weekly to monthly	31 (10.5)	4 (1.9)	123 (37.7)	18 (8.2)
	monthly to yearly	158 (53.6)	44 (21.0)	131 (40.2)	145 (66.2)
	yearly or less often	62 (21.0)	162 (77.1)	15 (4.6)	47 (21.5)
Time since last seizure	< 1 month	169 (56.5)	62 (29.5)	NA	10 (3.4)
	1–3 months	82 (27.4)	41 (19.5)	NA	5 (1.7)
	3–12 months	42 (14.0)	36 (17.1)	NA	20 (6.8)
	1–2 years	3 (1.0)	27 (12.9)	NA	69 (23.6)
	> 2 years	3 (1.0)	44 (21.0)	NA	188 (64.4)
Occurence of seizures	anytime	208 (69.3)	42 (24.6)	115 (31.8)	163 (54.3)
	daytime	54 (18.0)	25 (14.6)	119 (32.9)	77 (25.7)
	evening/night	6 (2.0)	47 (27.5)	32 (8.8)	40 (13.3)
	night while asleep	32 (10.7)	57 (33.3)	96 (26.5)	20 (6.7)
Precipitants before seizures		90 (30.2)	114 (53.8)	31 (8.5)	88 (29.3)
	alcohol	0/89	15/114 (13.2)	5/31 (16.1)	0/88
	fever	13/89 (14.6)	30/114 (26.3)	16/31 (51.6)	28/88 (31.8)
	stress/emotional stimuli	14/89 (15.7)	18/114 (15.8)	2/31 (6.5)	32/88 (36.4)
	lack of sleep	59/89 (66.3)	0/114	2/31 (6.5)	5/88 (5.7)
	other	9/89 (10.1)	62/114 (54.4)	6/31 (19.4)	23/88 (26.1)
	missing	1	0	0	0
History of febrile seizures		106 (35.7)	16 (7.6)	177 (48.4)	47 (15.7)
Past medical history		7 (2.3)	NA	37 (10.1)	33 (11.1)
	metabolic/cardiovascular	3/7 (42.9)	NA	3/37 (8.1)	2/33 (6.1)
	respiratory	1/7 (14.3)	NA	10/37 (27.0)	4/33 (12.1)
	infectious	3/7 (42.9)	NA	19/37 (51.4)	23/33 (69.7)
	other	0/7	NA	5/37 (13.5)	4/33 (12.1)
	missing	0	NA	0	0
Hospital admission		97 (32.4)	145 (68.4)	139 (38.1)	118 (39.3)
Past psychiatric history		162 (53.6)	18 (8.5)	62 (17.0)	20 (6.7)
Type	psychosis	72/162 (44.4)	0/18	17/62 (27.4)	6/20 (30.0)
	depression	13/162 (8.0)	0/18	2/62 (3.2)	7/20 (35.0)
	behavioural problems	19/162 (11.7)	4/18 (22.2)	22/62 (35.5)	3/20 (15.0)
	dementia	49/162 (30.2)	0/18	1/62 (1.6)	2/20 (10.0)
	mental retardation	33/162 (20.4)	16/18 (88.9)	29/62 (46.8)	14/20 (70.0)
	missing	0	0	0	0
Family history of epilepsy		101 (34.1)	57 (26.9)	107 (29.3)	88 (29.3)
Degree family member	first degree	47/100 (47.0)	39/57 (68.4)	43/101 (42.6)	11/86 (12.8)
	second degree	53/100 (53.0)	18/57 (31.6)	58/101 (57.4)	75/86 (87.2)
	missing	1	0	6	2
Mother not healthy during pregnancy		31 (12.0)	5 (2.4)	15 (4.1)	9 (3.0)
Schooling		275 (91.7)	143 (67.5)	273 (74.8)	192 (64.0)
Drop-out of school		55 (20.1)	59 (27.8)	162 (44.4)	118 (61.5)
Focal neurological signs on examination		23 (7.8)	NA	31 (8.5)	0
Diagnosis ILAE	generalised onset	240 (79.5)	149 (70.3)	288 (78.9)	277 (92.3)
	focal onset	61 (20.2)	61 (28.8)	77 (21.1)	23 (7.7)
	unclassified	1 (0.3)	2 (0.9)	0	0
Diagnosis Winkler et al.^21^	gen. seizures within a specific age range	175 (57.9)	119 (56.1)	109 (29.9)	127 (42.3)
	gen. seizures outside specific age range	47 (15.6)	30 (14.2)	152 (41.6)	67 (22.3)
	gen. seizures with diffuse brain damage	18 (6.0)	14 (6.6)	47 (12.9)	83 (27.7)
	gen. seizures with focal signs	57 (18.9)	47 (22.2)	45 (12.3)	23 (7.7)
	simple partial seizures	2 (0.7)	0	8 (2.2)	0
	complex partial seizures	2 (0.7)	0	4 (1.1)	0
	unclassified	1 (0.3)	2 (0.9)	0	0

The CT diagnoses of PWE can be found in Table 3; these were most commonly atrophy (8.2%) and post-ischaemic lesions (7.9%). PWE with focal onset seizures more commonly showed focal lesions (e.g. post-ischaemic lesions, NCC or tumours) than PWE with generalised onset seizures (Table 3). Overall, 728 of 941 (77.4%) CT scans were unremarkable, more among patients with generalised onset seizures than focal onset seizures (80.4% versus 64.9%).

**Table 3:** CT findings of persons with epilepsy, by type of seizure onset

	Overall (*n* = 941); n (%)^a^	Focal onset seizures (*n* = 168); n (%)	Generalised onset seizures (*n* = 771); n (%)	Unclassified seizures (*n* = 2); n (%)
Atrophy	77 (8.2)	10 (6.0)	67 (8.7)	0 (0)
Hydrocephalus	7 (0.7)	2 (1.2)	5 (0.6)	0 (0)
Post-ischaemic lesion	74 (7.9)	18 (10.7)	56 (7.3)	0 (0)
Post-haemorrhagic lesion	4 (0.4)	0 (0)	4 (0.5)	0 (0)
Traumatic lesion	3 (0.3)	3 (1.8)	0 (0)	0 (0)
Tumorous lesion	7 (0.7)	3 (1.8)	4 (0.5)	0 (0)
NCC	70 (7.4)	16 (9.5)	54 (7)	0 (0)
Other	55 (5.8)	19 (11.3)	36 (4.7)	0 (0)
Unremarkable	728 (77.4)	109 (64.9)	617 (80)	2 (100)

^a^More than one category could be selected

Chronic illnesses were infrequent apart from psychiatric disorders which were reported by 7%, 9% and 17% of patients in rural study sites and 54% of patients in the urban study site (Dar es Salaam). The most common psychiatric disorders were dementia, mental retardation, and psychotic episodes. Every third to fourth patient reported a family member with epilepsy, which in Tanzania (Dar es Salaam/Haydom) mostly was a first-degree family member. In comparison, in Uganda only 13% of patients had first degree family members with epilepsy. In the urban study site (Dar es Salaam), 92% of patients went to school, compared with 64% to 75% of patients at the rural study sites. Especially in Malawi and Uganda, many patients dropped out of school, often because of epilepsy (Table 2).

In the community-based studies less patients received ASM (Malawi: 68%, Uganda: 78%) compared with the hospital-based studies, where 90% (Dar es Salaam) and 99% (Haydom) of PWE were on ASM. Monotherapy with ASM was the most common form, which was present in almost all PWE, whose treatment modalities were recorded. A combination therapy was given to 11.6% and 3.1% of PWE in Dar es Salaam and Malawi, respectively (Table 4). The type of ASM varied according to availability, with carbamazepine being used by the majority of PWE in Tanzania (both Haydom and Dar es Salaam), phenytoin in Uganda and phenobarbital in Malawi. Information on treatment with ASM in the past was available for Dar es Salaam and Uganda only, with prior ASM in 27.2% and 10.3% of the patients, respectively. The most frequent cause for a change in ASM was not stock-out of medication but none-response to treatment and side-effects, mostly tiredness and/or dizziness (Table 4). Based on data from Haydom and Malawi where exact dosage for PWE on monotherapy was available, most PWE who were treated with phenobarbital, received a daily dose of 60 mg or less (38/53 (72%) in Haydom and 73/164 (45%) in Malawi). Furthermore, in Haydom 71/156 (45%) PWE who were treated with carbamazepine received 400 mg or less per day (Appendix Table 3).

**Table 4:** Treatment of persons with epilepsy from four different studies

		**Tanzania (Dar es Salaam)**	**Tanzania (Haydom)**	**Malawi**	**Uganda**
N		302	212	365	300
ASM		270 (89.7)	209 (98.6)	248 (67.9)	233 (77.7)
ASM type	monotherapy	243/268 (90.7)	209/209 (100)	222/229 (96.9)	233/233 (100)
	* carbamazepine*	134/268 (50.0)	156/209 (74.6)	7/229 (3.1)	31/233 (13.3)
	* phenobarbital*	103/268 (38.4)	53/209 (25.4)	201/229 (87.8)	55/233 (23.6)
	* phenytoin*	2/268 (0.7)	0/209	14/229 (6.1)	147/233 (63.1)
	* valproic acid*	4/268 (1.5)	0/209	0/229	0/233
	combination therapy	25/268 (9.3)	0/209	7/229 (3.1)	0/233
	* carbamazepine, phenobarbital*	13/268 (4.9)	0/209	0/229	0/233
	* carbamazepine, phenytoin*	4/268 (1.5)	0/209	2/229 (0.9)	0/233
	* carbamazepine, valproic acid*	1/268 (0.4)	0/209	0/229	0/233
	* phenobarbital, gabapentin*	1/268 (0.4)	0/209	0/229	0/233
	* phenobarbital, phenytoin*	6/268 (2.2)	0/209	5/229 (2.2)	0/233
	missing/unknown	2	0	19	0
Ever treated with other ASM		78 (27.2)	NA	NA	31 (10.3)
Reason for changing ASM	no response	9/24 (37.5)	NA	NA	17/31 (54.8)
	side effects	8/24 (33.3)	NA	NA	0/31
	stock-out	2/24 (8.3)	NA	NA	5/31 (16.1)
	other	5/24 (20.8)	NA	NA	9/31 (29.0)
	missing	54	NA	NA	0
Herbal treatment		159 (53.5)	105 (49.5)	304 (83.2)	108 (36.0)
Scarification		56 (19.2)	43 (20.3)	33 (9.0)	10 (3.3)

*ASM* Anti-seizure medication

In all three countries, a substantial proportion of patients received alternative non-ASM treatment. In Malawi five out of six patients received or had received herbal treatment for their seizures, and in Tanzania every fifth patient had scarification being performed as a cure for epileptic seizures.

## Discussion

In this study, we pooled the results of four studies on epilepsy in eastern Africa to describe the demographic, clinical, semiological, and neuroradiological characteristics, as well as treatment of PWE.

In the two community-based studies (Malawi and Uganda), a screening of people living in the target area was conducted first, comprising more than 100,000 people. Then, screen positives were seen by a neurologist to confirm diagnosis. Lifetime epilepsy prevalence reported in Malawi (1.1% [1.0–1.3%]) and Uganda (3.3% [3.1–3.4%]) were comparable, yet slightly higher than those from a previously published meta-analysis, which found a pooled lifetime prevalence of 0.7% (0.6–0.9%) for epilepsy in eastern Africa [23]. This could be due to our screening questionnaire, which may also have captured more patients with focal onset seizures, which are often not captured at all. Another explanation could be a higher prevalence of risk factors in our cohorts, e.g. NCC. In addition, other studies previously have reported even higher lifetime prevalence estimates for epilepsy of up to 8% [24, 25].

In general, studies estimate that in LMIC around 50% of PWE have secondary epilepsy [11]. In our study, we found a large proportion of people with seizure onset after adolescence which may indicate a predominance of secondary epilepsy. Unfortunately, we were not able to assess the exact proportion of persons with secondary epilepsy in our study. On the one hand because not many patients showed remarkable findings on CT scan, and for those who did, the temporal association could not be established. On the other hand, patients with obvious traumatic brain injury or perinatal hypoxic brain injury were excluded from further work-up due to study protocol, so that (CT) data on these aetiologies are not representative and most likely vastly underrepresented.

Nevertheless, CT data of almost 950 PWE were available, which to our knowledge represents the largest and most detailed analysis in sub-Saharan Africa. The rate of remarkable CT scan findings was highest in patients with focal onset seizures. Most common findings were neurocysticercosis-associated lesions, but interestingly, an even higher proportion of post-ischaemic lesions could be observed. This finding indicates that cerebrovascular events and post-stroke epilepsy have to be considered as an emerging aetiology for epilepsy in sub-Saharan Africa.

Another frequent CT pathology was cerebral atrophy, which is a common finding in PWE, but the significance and aetiology is hard to determine [26]. The rate of this pathology was higher in patients with general onset seizures, which is also known from previous studies, where patients with idiopathic generalised and genetic seizure syndromes showed diffuse and wide-spread alterations of brain morphology [26–28]. Detailed MRI analyses in these patients sometimes also reveal (wide-spread) cerebral malformations such as lissencephaly with pachygyria or microgyria potentially causing both focal and generalized seizures [29]. Further imaging studies in LMIC are needed to characterize the prevalence and significance of these findings.

The majority of PWE in the four studies had generalised onset seizures according to the ILAE criteria. In this analysis, we also assessed seizures according to the classification by Winkler et al. [22], which looks at epileptic seizures in low-resource settings in a more nuanced way, according to local contexts and needs, and tries to imply information on aetiologies and epilepsy syndromes based on seizure type and occurrence without accessory diagnostic. According to that adjusted classification by Winkler et al., most PWE with generalised seizure onset had generalised seizures within a specific age range, which represents the category in which a possible genetic background can be assumed. PWE of this group are suspected to suffer from idiopathic generalised epilepsies in a large proportion of cases. Consequently, our data suggest that despite a known high prevalence of risk factors for secondary epilepsy and previously reported high prevalence of focal epilepsies [30], genetic and idiopathic epilepsy syndromes also seem to play an important role in LMIC. This finding is of note, although a proportion of 30 to 58% among PWE might represent a certain overestimation in our study due to exclusion criteria for some types of secondary epilepsy as mentioned above. Nevertheless, as genetic origins are various and investigations in such a large and diverse cohort could reveal valuable results for both low-income and high-income countries, our results support the view, that it could be the time for initiating genetic epilepsy research in Africa [31] .

Despite a certain proportion of PWE with focal onset seizures in our analysis, most seizures were accompanied by a loss of consciousness and resulted in tonic–clonic seizures. Additionally, injuries during seizures were frequently observed, with a rate of up to 56% in the community-based studies. This is much higher than studies in high-income countries suggest [32, 33] and also is a possible explanation for high epilepsy-associated mortality rates in LMIC [34].

This lack of seizure control may display a lack in adequate ASM therapy. ASM treatment concerning both type and dosage differed substantially between and within countries. In Malawi and Uganda, phenobarbital and phenytoin were almost the only ASM prescribed; they were also still very common in Tanzania. In Tanzania, however, the majority of patients was treated with carbamazepine. Valproic acid was basically not available in any of the countries. These findings display the common picture of ASM treatment status in sub-Saharan Africa [35]. These findings, however, are not only limited to LMIC but can also be observed in high income countries [36]. Treatment choice in Africa very often depends on availability and cost of treatment and phenobarbital/phenytoin are often cheaper than carbamazepine or valproic acid [17]. Even though ASM are widely available, supply chain is often not sustained which results in stock-outs of medication [17, 37]. Nevertheless, stock-out of medication was not the main reason for changing ASM, but inadequate treatment effect and side-effects. Phenobarbital, in particular, is now rarely prescribed in high-income countries due to its relatively strong side effects and has been replaced by newer ASM [38]. Yet, it is still recommended by the World Health Organization (WHO) as first-line treatment in LMIC because of its cost advantage and broad spectrum of activity [35, 39]. Nevertheless, even in low-resource settings, phenobarbital has a higher risk to be withdrawn due to side-effects than carbamazepine, phenytoin, or valproic acid [39–41].

We found a quite large proportion of patients who had epileptic seizures more often than monthly, especially in the community-based studies, possibly reflecting the lower rates of ASM treatment compared to the hospital-based settings. However, we also found high frequencies of seizures even in patients receiving ASM, which may be due to barriers to accessing healthcare services, supply shortages, or the cost of ASM (mainly to hospitals and sometimes to patients, although ASM are often available free of charge), but also due to underdosage of ASM, which was common in our study. A large part of PWE in our pooled analysis received ≤ 60 mg phenobarbital or ≤ 400 mg carbamazepine per day.

Interestingly, seizure control varied considerably even among the two hospital-based studies, although both showed high treatment rates and treatment with carbamazepine was available. Haydom in rural Tanzania had a high proportion of patients with seizures only yearly or even less often, and no patients with seizures more frequent than monthly, despite underdosage was present in a certain proportion of patients. In contrast, PWE in urban Dar es Salaam showed high seizure rates, comparable to the community-based settings. This could be a result of the study settings, as PWE in Haydom were primarily recruited by a doctor and invited to follow-up visits in the clinic, probably getting access to ASM for the first time in some cases, resulting in good treatment effect, whereas patients in Dar es Salaam were recruited directly from the clinic. One could suspect a high proportion of patients with regular clinic visits due to difficulties to reach seizure control. This might be also reflected by the fact that this was the study site with the highest number of PWE receiving a combination therapy and having a history of prior change in ASM treatment. More studies comparing rural and urban healthcare settings are needed to assess the possible differences and contributing factors.

Many PWE across studies also received alternative treatment for their seizures. This shows the still very prevalent disbelieves about epilepsy as supernatural or witchcraft. In many regions of Africa, PWE are still being stigmatized which makes it difficult to analyse community-based prevalence and which may be the cause of the prevalent undertreatment of PWE.

Epilepsy has a big impact on social life as shown by the fact that many PWE dropped out of school, especially in Malawi and Uganda. In contrast, > 90% of PWE went to school in urban Dar es Salaam despite inadequate seizure control. One could hypothesize that disbelieves and stigmatization is less frequent in urban areas, although alternative non-ASM treatments were equally present in this group.

We report a rather large proportion of PWE who suffer from psychiatric disorders. Studies estimate that up to 50% of all PWE suffer from mental disorders, most commonly from depression, anxiety or dementia [42–45]. This proportion, however, is likely an overestimation as phenomena during seizures may be mischaracterised as mood disorders [46]. Those studies were mainly conducted in high-income countries, and only one was from Sierra Leone. For sub-Saharan Africa, data are still scarce. In our analysis, the proportion of patients with psychiatric disorders differed substantially between studies, which may be due to different assessors, as the questionnaire was the same across countries. Interestingly, prevalence of psychiatric disorders was highest in the urban study site of Dar es Salaam. Differences in education and work as well as different degrees of stigmatization of psychiatric disorders may be a possible explanation. In the four studies, only a small proportion of patients suffered from chronic diseases other than psychiatric illnesses, which is not unusual in sub-Sahara Africa, mostly because diseases can be diagnosed with less certainty.

### Strengths and limitations

This pooled collection of demographic, clinical, semiological, neuroradiological and therapeutic data on PWE in three countries of East Africa is exceptional and represents sustained work of over 10 years. We give data on epilepsy prevalence proportions derived from two large community-based studies, including screening of over 100,000 people, and describe the different characteristics and treatment of nearly1200 PWE including CT examinations in almost 950 cases, which is unique within the sub-Saharan African setting.

Consequently, this study represents an important piece of work and adds substantially to the body of literature on epilepsy globally, and specifically to the evaluation and understanding of the burden of epilepsy and the persisting treatment gaps in sub-Saharan Africa. This has great policy relevance, not only for the respective countries, i.e. Tanzania, Uganda and Malawi, but also at a global level, considering both, the burden of epilepsy, and the growing burdon of neurological disorders in LMIC in general [47, 48]. In addition, results from the present analysis may also support the WHO Intersectoral Global Action Plan on Epilepsy and other Neurological Disorders which is currently being drafted [19].

However, our analysis also suffers from several limitations.

First, examinations were performed by different personnel and translation of the questionnaire to different languages had to be conducted. This may have influenced the results. Nevertheless, studies applied the same approach to history taking and investigations for the diagnosis of epilepsy. Consequently, the data we focused on in our analyses, such as demographic factors or data on seizure onset and frequency were probably less affected.

As all scans underwent examination by the same radiologist (VR), we consider CT findings reliable and comparable among cohorts. Nevertheless, CT machines and quality of CT scans at different study sites varied considerably and are not comparable to high-income settings. Thus, CT findings may not be as accurate as desired. Additionally, MRI imaging for a more detailed assessment was basically not available.

Due to the studies’ NCC specific settings, investigations focused on evaluation of cryptogenic focal epilepsy and excluded e.g. patients with known traumatic brain injury, leading to a possible overestimation of non-focal epilepsies. A broader approach might have given a more detailed insight in epilepsy aetiologies.

In terms of seizure semiology, we did not assess focal onset signs and symptoms in detail, and therefore were not able to evaluate data with regard to possible epilepsy locations and syndromes such as e.g. temporal lobe epilepsy. Future analyses should give details on awareness during focal seizures, as well as on quality of non-motor symptoms, e.g. autonomic, sensory, cognitive, and emotional symptoms respectively. Additionally, as not all patients were assessed by neurologists, some focal (onset) signs or uncommon seizure semiologies might have been missed in our studies, contributing to a potential under-estimation of focal epilepsies. As accessory diagnostics are limited in LMIC settings, a distinctive and precise history taking might be the best chance to get a more detailed picture of possible epilepsy syndromes and aetiologies.

Another limitation of our study was the mix of hospital-based and community-based studies in our pooled analysis, which probably lead to heterogeneous patient cohorts and occurring selection bias as discussed above. Nevertheless, if one is aware of the possible problems, this heterogeneity also offers the possibility to observe and analyse the possible differences of study population characteristics. Nevertheless, stigma and only slight signs/symptoms might have influenced the recruitment process in the community-based studies [9]. We tried to constantly indicate the differences between hospital-based and community-based studies throughout our manuscript and therefore consider this pooled analysis appropriate and valuable.

## Conclusion

In this large and comprehensive study, data on demographic, semiological, clinical, neuroradiological and therapeutic findings of four studies among PWE in eastern Africa were pooled. Overall, epilepsy is common in sub-Saharan Africa and PWE suffer from high seizure rates and subsequent injuries as well as from socio-economic consequences, such as drop out from school. ASM treatment rates are rather low, especially in rural areas, and underdosage is common. Secondary epilepsy plays an important role with CT imaging revealing both, neurocysticercosis-associated and post-ischaemic lesions as the main findings in our study setting. Nevertheless, a large proportion of PWE can also be assumed to suffer from genetic epilepsy syndromes, although study setting might have overestimated this group.

This pooled analysis contributes to the evaluation and understanding of the burden of epilepsy and the persisting treatment gap in sub-Saharan Africa. Our results demonstrate the need of structural programmes, addressing both, acquisition of material resources and transfer of expert knowledge, for adequate identification, education, and treatment of PWE.

## Supplementary Information

**Additional file 1: Appendix Information 1.** Methodology of the included studies. **Appendix information 2.** Ethical clearance obtained. **Appendix Table 1.** Epilepsy screening questionnaire Malawi. **Appendix Table 2.** Epilepsy screening questionnaire Uganda. **Appendix Table 3.** Type and dosage of ASM among patients treated with monotherapy^¥^.

## Data Availability

The datasets generated and/or analysed during the current study are not publicly available because further publications are still being analysed from the data. However, data are available from the corresponding author on reasonable request.

## Abbreviations

PWE: Persons with epilepsy
CT: Computed tomography
IQR: Interquartile range
NCC: Neurocysticercosis
ASM: Anti-seizure medication
LMIC: Low-income and middle-income countries
MRI: Magnetic resonance imaging
WHO: World Health Organization
