# Correction: Characteristics of people with epilepsy in three Eastern African countries – a pooled analysis

**DOI:** 10.1186/s12883-022-03034-0

**Published:** 2022-12-23

**Authors:** Dominik Stelzle, Joyce Kaducu, Veronika Schmidt, Tamara M. Welte, Bernard J. Ngowi, William Matuja, Gabrielle Escheu, Peter Hauke, Vivien Richter, Emilio Ovuga, Bettina Pfausler, Erich Schmutzhard, Action Amos, Wendy Harrison, Luise Keller, Andrea S. Winkler

**Affiliations:** 1 Center for Global Health, Department of Neurology, School of Medicine, Technical University of Munich, Ismaninger Strasse 22, 81675 Munich, Germany; 2 Ministry of Health, Kampala, Republic of Uganda; 3 Centre for Global Health, Institute of Health and Society, University of Oslo, Oslo, Norway; 4 Department of Neurology, University Hospital Erlangen, Erlangen, Germany; 5 National Institute for Medical Research, Muhimbili Medical Research Centre, Dar es Salaam, Tanzania; 6 University of Dar Es Salaam, Mbeya College of Health and Allied Sciences, Mbeya, Tanzania; 7 Department of Neurology, Muhimbili University of Health and Allied Sciences, Dar es Salaam, Tanzania; 8 Department of Neurology, Kliniken Ostallgaeu-Kaufbeuren, Kaufbeuren, Germany; 9 Department of Radiology, University Hospital Tuebingen, Tuebingen, Germany; 10 Department of Mental Health, University of Gulu, Gulu, Uganda; 11 Department of Neurology, Medical University of Innsbruck, Innsbruck, Austria; 12 National Epilepsy Association Malawi, International Bureau of Epilepsy, Lilongwe, Malawi; 13 Department of Infectious Disease Epidemiology, Imperial College London, London, UK; 14 Department of Neurology, School of Medicine, Technical University of Munich, Munich, Germany

**Correction: BMC Neurol 22, 321 (2022)**

**https://doi.org/10.1186/s12883-022-02813-z**

Following publication of the original article [1], the authors would like to update the Funding section.

The updated Funding is provided below and the changes have been highlighted in **bold**.

## Funding

Open Access funding enabled and organized by Projekt DEAL. This study was funded by the German Research Foundation (DFG) within the research grant (WI 3427/1–1) “Neurocysticercosis in sub-Saharan Africa” and by the Bill and Melinda Gates Foundation within the research grant (1017886) “Integrated Control of Cysticercosis in sub-Saharan Africa”. **Dominik Stelzle was supported by the German Federal Ministry of Education and Research, Grant 01KA2112B, within the Cysticercosis Network of Sub-Saharan Africa (CYSTINET-Africa).** The funders had no role in the detailed study design, data collection and analysis, decision to publish, or preparation of the manuscript.

The original article [1] has been updated.
