# Supplementary material for: Characteristics of people with epilepsy in three Eastern African countries – a pooled analysis
Source: BMC Neurol. 2022 Aug 26;22:321. doi: 10.1186/s12883-022-02813-z (PMC9414166; doi:10.1186/s12883-022-02813-z)
Supplement: Supplementary file 1 — Additional file 1: Appendix Information 1. Methodology of the included studies. Appendix information 2. Ethical clearance obtained. Appendix Table 1. Epilepsy screening questionnaire Malawi. Appendix Table 2. Epilepsy screening questionnaire Uganda. Appendix Table 3. Type and dosage of ASM among patients treated with monotherapy¥. [file 12883_2022_2813_MOESM1_ESM.docx]

- Supporting Information -

**Characteristics of People with Epilepsy in Three Eastern African Countries – a Pooled Analysis**

Dominik Stelzle MD^1^*, Joyce Kaducu PhD^3^*, Veronika Schmidt PhD^1,4^, Tamara M Welte MD^1,5^, Bernard J Ngowi PhD^6,7^, William Matuja PhD^8^, Gabrielle Escheu MD^9^, Peter Hauke MD^9^, Vivien Richter MD^10^, Emilio Ovuga PhD^11^, Bettina Pfausler PhD^12^, Erich Schmutzhard PhD^12^, Action Amos PhD^13^, Wendy Harrison PhD^14^, Luise Keller MD^15^*, Andrea S Winkler PhD^1,4^*

**Short title:** Epilepsy in eastern Africa

^1^ Center for Global Health, Department of Neurology, School of Medicine, Technical University of Munich, Germany

^3^ Ministry of Health, Republic of Uganda

^4^ Centre for Global Health, Institute of Health and Society, University of Oslo, Norway

^5^ Department of Neurology, University Hospital Erlangen, Germany

^6^ National Institute for Medical Research, Muhimbili Medical Research Centre, Dar es Salaam Tanzania

^7^ University of Dar es Salaam, Mbeya College of Health and Allied Sciences, Mbeya Tanzania

^8^ Department of Neurology, Muhimbili University of Health and Allied Sciences, Dar es Salaam, Tanzania

^9^ Department of Neurology, Kliniken Ostallgaeu-Kaufbeuren, Germany

^10^ Department of Radiology, University Hospital Tuebingen, Tuebingen, Germany

^11^ Department of Mental Health, University of Gulu, Uganda

^12^ Department of Neurology, Medical University of Innsbruck, Austria

^13^ National Epilepsy Association Malawi, International Bureau of Epilepsy

^14^ Department of Infectious Disease Epidemiology, Imperial College London, United Kingdom

^15^ Center of Neurology, Berlin, Germany

**Corresponding author:**

Prof. Andrea S Winkler MD, PhD

Department of Neurology

Klinikum rechts der Isar

Technical University Munich

Ismaninger Strasse 22

81675 Munich, Germany

tel.: +49/89/41406954

email: andrea.winkler@tum.de

*Appendix Information 1. Methodology of the included studies*

Recruited were consecutive patients attending mental health clinic at both sites (≥6 years in Haydom and ≥11 years in Dar es Salaam). CT scans were performed on all patients who fulfilled the inclusion criteria in Haydom and due to financial reasons, a random subset of 302 PWE in Dar es Salaam.

The two other studies included community-based screenings of 107,898 individuals in total, one in rural northern Uganda and the other one in rural southern Malawi. The aim of the study in Malawi was a risk-assessment of neurological side effects due to NCC following mass drug administration (MDA) of praziquantel for the control of schistosomiasis. From October 2012 to December 2013, community-based door-to-door screening was performed in rural Balaka district with assessment of nearly the entire population of the areas Chiyendausiku, Kalembo and Mbera. Areas were selected because of geographic presence of porcine cysticercosis as sentinel for human disease. Prior to MDA, screening for epileptic seizures was performed among almost 70,000 people, using a 15-item questionnaire (appendix table 1). Overall, 3100 people screened positive for epileptic seizures of which 455 could be diagnosed with epilepsy. All patients <6 years only suffering from seizures in childhood were excluded, accounting for 440 people. Lost to follow-up or refusal to study occurred in 75 cases, resulting in 365 PWE. Due to financially limited resources, only 127 selected PWE could receive a CT scan, focusing on recent epilepsy onset and serological test results for *T. solium* cysticercosis.

The aim of the study in Uganda was an assessment of NCC prevalence in a rural PWE population in three districts in northern Uganda (Adjumani, Gulu and Moyo). From May 2010 to March 2011, a two-step random-cluster community-based door-to-door study was conducted. Study sites were selected due to geographic presence of porcine cysticercosis as sentinel for human disease. Of nearly 40,000 people, 1254 individuals ≥ 12 years were screened positive for epileptic seizures by a 9-item questionnaire (appendix table 2). After application of inclusion and exclusion criteria as described above, 1083 were eligible for further assessment. Because of financial issues only 300 were selected for further clinical evaluation. All 300 PWE were offered serological testing for *T. solium* cysticercosis and if positive, the patient was offered a CT scan. Additionally, all serologically negative patients with seizure onset in the previous 5 years and a 14 of 197 PWE with seizure onset longer than 5 years prior to the study, received a CT scan.

In all studies, diagnosis of epilepsy required two or more afebrile epileptic seizures in the absence of acute metabolic disorders or withdrawal of drugs or alcohol. Only patients fulfilling this definition and giving consent to the different study procedures were included in the studies. Due to study settings aiming to assess NCC prevalence proportions, patients with obvious non-infectious aetiologies such as perinatal hypoxic brain injury, traumatic brain injury or a history of chronic alcohol abuse were excluded from further study assessment. Recruitment of children depended on a) the need to exclude children suffering from febrile seizures and b) on the statement of the local ethics committee on clinical indication for undergoing radiation from computed tomography (CT) examination. Neurological examination was performed on all recruited PWE and completion of a thorough questionnaire on medical history focusing on seizure history and semiology was required.

*Appendix information 2. Ethical clearance obtained*

Tanzania (both for Dar es Salaam and Haydom): National Institute of Medical Research in Dar es Salaam (Ref. No.MU/DRP/AEC/VOL. XIII/64); Directorate of Research and Publications, Muhimbili University of Health and Allied Sciences (MUHAS), Dar es Salaam (Ref. No.: MU/DRP/REC/Vol.I/36, MU/RP/AEC/Vol.XII/86 and MU/DRP/AEC/Vol.XVI/91).

Germany: The Ethics Committee of the Technical University of Munich (TUM) approved the study in Malawi (Ref. No. 3088/10). For the study in Tanzania the Ethics Committee of Ludwig-Maximilians University (LMU) Munich, Germany stated LMU clearance in Germany not to be necessary (correspondence from 23082008).

Malawi: Malawi National Health Science Research Committee, NHSRC Ref. No. 910; Imperial College Research Ethics Committee, Ref. No. ICREC_11_3_6.

Uganda: Uganda National Committee for Science and Technology, UNCST Ref. No. 543.

Clinical sites: Tanzania: Dar es Salaam Muhimbili hospital; Haydom Lutheran Hospital, Haydom, Tanzania

Appendix Table 1. Epilepsy screening questionnaire Malawi

| 1. | Have you ever lost consciousness or fallen due to lost consciousness? |
| --- | --- |
| 2. | Have you ever been told that while you were unconscious your arms and legs shake or stretch out? |
| 3. | Have you had attacks in which you fall and bite your tongue or lost control of your bladder or bowels? |
| 4. | Have you had uncontrollable attacks of shaking or trembling in one arm or leg or in the face without losing consciousness? |
| 5. | Have you had attacks in which you lose contact with the surroundings without losing consciousness? |
| 6. | Have you ever been told that you had episodes of strange behaviour without remembering it? |
| 7. | Have you ever been told that you had epilepsy or epileptic seizures? |
| 8. | Did you/your child have seizures between one month and 7 years of age? |
| 9. | Was there a fever with all of the attacks? |
| 10. | Did the seizures continue after the age of 7? |
| 11. | How old were you when you had your first attack (enter YY:MM)? |
| 12. | Have you had an attack in the last 4 weeks? |
| 13. | How long ago in months was your last attack? |
| 14. | How often do you have these attacks?  1 Daily 2 Once a week or more but less than daily 3 Once a month or more but less than once a week 4 Once every six months or more but less than once a month  5 Once a year or more but less than once every six months  6 Less than once a year 7 Not applicable |
| 15. | Do you take antiepileptic medication? |

Appendix Table 2. Epilepsy screening questionnaire Uganda

| 1. | Have you ever lost consciousness or fallen due to lost consciousness? |
| --- | --- |
| 2. | Have you ever been told that while you were unconscious your arms and legs shake or stretch out? |
|  | Have you ever had attacks in which you fell and bit your tongue or lost control of your bladder or bowels? |
|  | Have you ever had uncontrollable attacks of shaking or trembling in one arm or one leg, or in the face without losing consciousness? |
|  | Have you ever experienced attacks of numbness, tingling in one arm or one leg without losing consciousness? |
|  | Have you ever had attacks in which you lose contact with the surroundings without losing consciousness? |
|  | Have you ever had attacks of losing awareness that was associated with a feeling of vagueness, unreality or dreaminess or experience of abnormal smells, sounds, or vision without losing or before loss of consciousness? |
|  | Have you ever been told that you had episodes of strange behaviour without remembering it? |
|  | Have you ever been told that you have or had epilepsy or epileptic seizures? |

Appendix Table 3. Type and dosage of ASM among patients treated with monotherapy­^¥^

|  | | Tanzania  (Haydom) | Malawi |
| --- | --- | --- | --- |
| ASM monotherapy^⸸^ | | 209/209 (100) | 222/229 (96.7) |
| Phenobarbital | 30mg | 2/53 (4) | 41/164 (25) |
|  | 60mg | 36/53 (68) | 32/164 (20) |
|  | 90mg | 14/53 (26) | 43/164 (26) |
|  | 120mg | 1/53 (2) | 48/164 (29) |
|  | NA | 0 | 52 |
| Carbamazepine | 200mg | 5/156 (3) | 1/1 (100) |
|  | 400mg | 66/156 (42) | 0 |
|  | 600mg | 14/156 (9) | 0 |
|  | 800mg | 71/156 (46) | 0 |
|  | NA | 0 | 6 |
| Phenytoin | 50mg | NA | 2/14 (14) |
|  | 100mg | NA | 7/14 (50) |
|  | 150mg | NA | 2/14 (14) |
|  | 300mg | NA | 3/14 (21) |
|  | NA | NA | 0 |

¥ No data were available for Tanzania (Dar es Salaam) and Uganda

⸸ The denominator in the first row are all patients who are on ASM (including combination therapy)
